# Assessing phenotypic diversity and trait relationships within (*Syzygium cumini* (L.) Skeels) using morpho-biochemical traits

**DOI:** 10.7717/peerj.21302

**Published:** 2026-05-25

**Authors:** Anshuman Singh, Ravi Kumar Singh, Anju Bajpai, Nitin Kumar Singh, Shiwanand Pandey, Akath Singh, Damodaran Thukkaram

**Affiliations:** ICAR- Central Institute for Subtropical Horticulture, Lucknow, Uttar Pradesh, India

**Keywords:** Jamun, Morpho-biochemical traits, Phenotypic variability, Trait correlations, MGIDI index

## Abstract

**Background:**

Jamun (*Syzygium cumini* (L.) Skeels) is a multipurpose fruit tree with notable nutritional, medicinal, and ecological potential. Uttar Pradesh, a major jamun growing state in India, harbours considerable variability in jamun, which remains underexplored.

**Methods:**

To assess phenotypic variability and identify superior types, 123 genotypes of jamun were evaluated using morphological descriptors. Biochemical attributes were also assessed for a subset of 15 genotypes from the full panel.

**Results:**

Significant variation was observed for most qualitative descriptors, with clear differences in tree growth and fruit morphology. Quantitative traits such as fruit weight (2.04–17.66 g), pulp content (61.12–94.38%), pulp: seed ratio (1.60–18.85) and total soluble solids (TSS, 10.64–19.96° Brix) exhibited wide ranges. Biochemical characterization of a subset of genotypes further revealed notable variation in ascorbic acid, anthocyanin content, and antioxidant-related traits, with genotypes Gn-10, Gn-11 and Br-17 showing favourable biochemical profiles. Correlation analysis indicated strong positive associations among fruit size traits (fruit length, diameter, and weight), whereas TSS showed negative correlations with fruit size and pulp-related traits. Cluster analysis based on morphological traits identified two major phenotypic groups, with the majority of genotypes forming a dominant cluster (*n* = 114) and a smaller group (*n* = 9) showing greater phenotypic divergence. Multitrait Genotype–Ideotype Distance Index identified Gn-18, Br-2, Br-14, Gn-11 and Un-6 as the most ideotype-consistent genotypes, based on fruit weight, pulp content and TSS.

**Conclusions:**

While the present study highlights phenotypic diversity among the studied seedling-origin jamun trees, multi-year evaluations along with molecular characterization are needed to better elucidate the extent of diversity, the genetic basis of the observed trait differences, and the relationships among genotypes.

## Introduction

Despite being largely neglected in research and trade, the underutilized crops remain reliable sources of food, income, and medicine (Chandra et al., 2020; Dar et al., 2022), and hold substantial potential for addressing hunger and malnutrition, diversifying agri-food systems, conserving agro-biodiversity, and adapting to the impacts of climate change (Baldermann et al., 2016). Jamun or Indian blackberry (*Syzygium cumini* (L.) Skeels, family Myrtaceae), an underutilized fruit tree of Indian origin, is widely distributed across the Indian subcontinent, Southeast Asia, East Africa, and several other countries of world (Madani et al., 2021; Khadivi et al., 2023). A multipurpose species in traditional agroforestry systems, its fruits are consumed fresh or processed into value-added products such as jam, squash, and syrup (El-Safy et al., 2023). Along with custard apple, Indian jujube, jackfruit, and tamarind, jamun is considered a promising fruit for addressing nutrition insecurity in India (Dandin et al., 2019). It exhibits resilience to abiotic stresses such as drought, salinity and waterlogging. Reforesting degraded lands with jamun can greatly contribute to biodiversity conservation, carbon sequestration, ecological sustainability and enhanced food production (Sarvade et al., 2016).

Ripe jamun fruits are rich in dietary fiber, vitamins, minerals and phenolic compounds (Tak et al., 2022). Both the fruit pulp and seed contain anthocyanins, flavonoids and polyphenols that exhibit antioxidant, anti-proliferative and chemo-preventive properties (Chhikara et al., 2018). Their strong therapeutic potential in managing diabetes and other metabolic disorders is well documented (Gajera et al., 2017; Sane, Rekha & Tripathi, 2025). Despite these benefits, jamun remains an underutilized fruit crop in India, and its genetic diversity is increasingly threatened by urbanization and habitat loss (Sane, Rekha & Tripathi, 2025). Given the increasing demand for jamun fruits and seeds for nutritional and therapeutic applications, systematic documentation of existing phenotypic diversity is essential to support conservation priorities and support future improvement efforts (Khadivi et al., 2023). Although a few improved cultivars and management practices have been developed, jamun cultivation and trade in India remain largely localized (Singh et al., 2011). Achieving wider commercialization in domestic and international markets will depend on the systematic identification of promising germplasm possessing desirable combinations of traits (Singh et al., 2019; Madani et al., 2021).

The collection, characterization, and conservation of diverse genetic resources are crucial for developing high-yielding jamun cultivars with superior fruit quality (Ahmad et al., 2012; Singh et al., 2020a; Singh et al., 2020b). Recent investigations into jamun genetic resources have employed diverse approaches, including evaluations based solely on morphological traits (Prakash, Maurya & Singh, 2010; Srivastava, Rai & Kumar, 2010; Devi, Swamy & Naik, 2016; Swamy, Anushma & Jagadeesha, 2017; Plathia et al., 2018), integrated morpho-biochemical assessments (ud Din et al., 2020; Dissanayake et al., 2022; Kumar et al., 2025), combined morpho-molecular analyses (Singh et al., 2022; Sharma et al., 2023; Uddin et al., 2024) and biochemical-molecular analysis (Khan et al., 2023). Nonetheless, these studies are constrained by small sample sizes, and restricted geographic coverage. While our work is also geographically restricted to Uttar Pradesh state of India, it improves upon earlier studies by sampling a substantially larger number of seedling origin jamun trees (*n* = 123) across multiple districts, thereby providing a more comprehensive characterization of phenotypic variability within this region. Wide variation in soil types, rainfall, temperature, and other agro-climatic factors has contributed to the development of diverse seedling populations of jamun in Uttar Pradesh state of India. These populations, which exhibit substantial differences in fruit and seed attributes, provide valuable opportunities for documenting phenotypic variation and identifying promising germplasm based on desirable trait combinations (Singh et al., 2011). However, systematic studies on jamun in this region remain scarce (Patel et al., 2005), highlighting the need for comprehensive characterization of existing phenotypic diversity to contribute to informed germplasm conservation strategies and facilitate evidence-based prioritization of promising genotypes. Accordingly, the present work constitutes a region-specific phenotypic survey designed to systematically characterize phenotypic variation among seedling origin jamun trees.

In view of these gaps, the present study was carried out to collect and phenotypically characterize diverse jamun germplasm from Uttar Pradesh, India, with focus on assessing the extent of phenotypic variability in morphological and fruit quality traits. Emphasis was placed on identifying superior and contrasting genotypes with desirable attributes such as higher fruit weight and more pulp content. The outcomes of this study are expected to provide a scientific basis for targeted genetic conservation and prioritization, thereby facilitating the development of superior cultivars and supporting sustainable cultivation and commercialization of jamun.

## Materials and Methods

### Study area and experimental material

Surveys were conducted during 2022–2024 across Ayodhya, Barabanki, Gonda, Hardoi, Lucknow, Siddharth Nagar and Unnao districts of Uttar Pradesh, India to collect and characterize seedling-origin jamun genotypes using a set of qualitative and quantitative phenotypic descriptors (Raza et al., 2017). The study districts have a subtropical climate (NICRA, 2025). Seedling-origin jamun trees in this region exhibit considerable variability in fruit quality attributes, providing opportunity for phenotypic diversity assessment and the identification of elite genotypes (Singh et al., 2011). Surveys were conducted during June–July, the peak fruit-ripening period for jamun in the study area (Patel et al., 2005). Jamun trees of comparable age (30–35 years) were selected (Khan, Vaishali & Sharma, 2010), with tree age estimated based on information provided by local inhabitants (Sunil et al., 2008). It was ensured that trees sampled within each district are morphologically distinct (ud Din et al., 2020) and sufficiently physically isolated (Khadivi et al., 2020) to minimize the likelihood of sampling phenotypically similar genotypes. All sampled trees were under unmanaged conditions (Cristóbal-Pérez et al., 2022). Geographical coordinates for each tree are provided in Table S1. In total, 123 putative genotypes (hereafter referred to as 123 genotypes) were evaluated to assess phenotypic diversity, and to identify promising genotypes based on desirable pomological traits.

### Observations recorded

Observations on tree, leaf, and fruit traits were recorded for 123 jamun genotypes, with each tree considered as an experimental unit (ud Din et al., 2020). Fully developed leaves and ripe fruits (*n* = 10) were randomly sampled from each tree (experimental unit), and were treated as within-tree subsamples. A total of 24 descriptors, comprising 12 qualitative and 12 quantitative traits, were used for characterization (PPVFRA, 2025). To minimize maturity-related variation, fruits were harvested at comparable physiological maturity, determined by fruit colour, and ease of detachment. Fifteen (*n* = 15) jamun genotypes were also assessed for fruit biochemical traits. These genotypes were randomly selected represent the observed phenotypic variation among the 123 genotypes.

### Qualitative traits

Jamun genotypes were characterized using qualitative descriptors of tree, leaf, and fruit traits. Tree growth was characterized using growth habit (spreading, semi-spreading, upright) and foliage density (sparse, dense). Leaf characterization included five traits: apex (acute, acuminate), base (acute, round), mature leaf colour (green, dark green), petiole length (short, medium, long), and leaf length-to-width ratio (low, high). Fruit descriptors included mature fruit colour (purple red, dark purple, purple black), fruit shape (oblong, elliptic, ovoid, round), fruit apex (flat, depressed, round), stalk end (nipple-shaped, flattened, depressed) and pulp colour (cream white, purple white).

### Quantitative traits

Leaf length and width were measured on ten fully developed leaves (*n* = 10) using a measuring scale. Fruit and seed physical attributes were recorded on ten within-tree subsamples per genotypes (*n* = 10). Fruit and seed length and diameter were determined with a digital caliper (Mitutoyo, Japan), and their weights were recorded using an electronic balance of 6 kg capacity and 0.1 g accuracy (TS-200, India). The caliper was calibrated using gauge blocks to verify measurement accuracy prior to actual measurements. Pulp content (PC) was calculated using the formula: PC (%) = (pulp weight/fruit weight) × 100. The pulp: seed ratio (PSR) was computed as the ratio of fruit weight to seed weight.

### Fruit biochemical attributes

A subset of 15 phenotypically diverse genotypes, randomly selected from the 123 evaluated genotypes, was further examined for biochemical traits using the methods outlined below. All biochemical estimations were performed on three within-tree subsamples (biological replicates) per genotype to account for within-tree variation.

#### Total soluble solids, titrable acidity and juice pH

Total soluble solids (TSS) were measured using a digital refractometer (Atago, Japan), calibrated with distilled water (0.0° Brix) and standard sucrose solutions (30.0 and 50.0° Brix) prior to measurements. A drop of freshly extracted juice was placed on the prism, and values were recorded as degrees Brix (^∘^B). Titratable acidity was determined by homogenizing fresh fruit pulp (0.5 g) in deionized water and making the final volume up to 50 ml. An aliquot of the extract was titrated against 0.1 N sodium hydroxide solution using phenolphthalein as an indicator until a persistent pale-pink endpoint was observed (AOAC, 2002). Juice pH was measured using a digital pH meter (Hanna Edge, Romania), calibrated with standard buffer solutions of pH 4.0 and 7.0 prior to measurement. The probe was immersed directly in freshly extracted juice to record the pH values. TSS and pH measurements were carried out at controlled temperature (25 ± 2 °C) to minimize temperature-related measurement bias.

#### Ascorbic acid

Ascorbic acid was determined following the method of Jones & Hughes (1983). The dye solution was standardized against an ascorbic acid standard prior to analysis. Fresh fruit pulp (0.5 g) was homogenized and diluted to 50 ml with 3% (w/v) metaphosphoric acid. A 10 ml aliquot was titrated with 2,6-dichlorophenolindophenol dye until the appearance of a persistent pink endpoint (15 s). The titration volume was recorded and used to calculate ascorbic acid content, expressed as mg per 100 g fresh weight (mg 100 g^−1^ FW).

#### Total anthocyanin content

Total anthocyanin content was determined following the method of Lees & Francis (1972). Fruit pulp (5.0 g) was extracted with ethanolic HCl and stored overnight at 7 ± 1 °C. The extract was then centrifuged at 5,600 × g for 10 min, and absorbance was measured at 535 nm using a UV–visible spectrophotometer (Genesys 20, Thermo Fisher Scientific, Waltham, MA, USA). Total anthocyanin content was expressed as mg 100 g^−^^1^ fresh weight (FW).

#### Total flavonoid content

Total flavonoid content was determined using the aluminum chloride colourimetric method (Chang et al., 2002). Fruit pulp (5 g) was extracted with 20 ml of 80% methanol and centrifuged at 6,000 × g for 15 min at 4 °C using a refrigerated centrifuge (iFuge UC02R, Neuation, India). An aliquot (0.5 ml) of the extract was mixed with 2 ml of distilled water, 3 ml of 5% sodium nitrite, 0.3 ml of 10% aluminum chloride, and 2 ml of 1 M sodium hydroxide, followed by incubation for 30 min. Absorbance was measured at 415 nm against a blank using a UV–visible spectrophotometer (Genesys 20, Thermo Fisher Scientific, Waltham, MA, USA), and total flavonoid content was expressed as mg quercetin equivalents per gram fresh weight (mg QE g^−^^1^ FW). The calibration curve used for quantification is presented in Fig. S1A.

#### Total phenol content

Total phenolic content was determined using the procedure described in McDonald et al. (2001). Fresh fruit pulp (1.0 g) was extracted using 10 ml methanol: water (50:50 v/v). An aliquot (0.5 ml) of the diluted extract (1:10) or gallic acid standard was mixed with 5 ml of Folin–Ciocalteu reagent (1:10 diluted with distilled water) and 4 ml of 1 M Na_2_CO_3_, and the reaction mixture was allowed to stand for 15 min at room temperature. Absorbance was measured at 765 nm against a blank using a UV–visible spectrophotometer (Genesys 20, Thermo Fisher Scientific, Waltham, MA, USA). Total phenolic content was expressed as mg gallic-acid equivalents per 100 g fresh weight (mg GAE 100 g^−^^1^ FW). The calibration curve used for quantification is presented in Fig. S1B.

#### Radical scavenging activity

Radical scavenging activity in terms of antioxidant capacity was determined by assessing free radical-scavenging effect on 2,2-diphenyl-1-picrylhydrazyl (DPPH) radicals following the method described in De Ancos, González & Cano (2000). Fresh fruit pulp (1 g) was extracted with 5 ml of methanol, followed by centrifugation at 6,000 × g for 15 min at 4 °C, using a refriferated centrifuge (Neuation iFuge UC02R, India). An aliquot of the extract (10 µl) was mixed with 3.9 ml methanolic DPPH solution (0.025 g l^−^^1^) and 90 µl distilled water. The mixture was vortexed (Simco) and incubated in the dark for 30 min, after which absorbance was recorded against the blank at 515 nm using a UV-visible spectrophotometer (Genesys 20, Thermo Fisher Scientific, Waltham, MA, USA). Scavenging activity was expressed as percentage inhibition of DPPH radicals relative to the control solution (%).

#### Statistical analysis

This study evaluated 123 jamun genotypes collected from seven districts of Uttar Pradesh, India with each genotype originating from only one district. Different genotypes were sampled each year (2022–2024) across the surveyed districts, and no repeated measurements were taken from the same genotype across years. In each year, all traits were recorded on ten leaves or fruits sampled per tree (*n* = 10) and treated as within-tree subsamples. Similarly, biochemical attributes were measured on a subset of 15 randomly selected genotypes from the full panel using three within-tree subsamples per genotype. Consequently, the dataset was unbalanced and did not conform to a classical randomized block design. Therefore, the study was framed as a phenotypic diversity assessment and analytical methods appropriate for unbalanced datasets were applied to delineate existing phenotypic variation. Because genotypes were not repeated across years, year effects and genotype × year interactions were not estimable. Analyses therefore used tree-level phenotypic means, with inferences restricted to phenotypic variation. Qualitative phenotypic traits were analyzed as categorical variables, with category-wise frequencies and proportions computed for each trait. Departures from equal category representation were tested using chi-square goodness-of-fit tests comparing observed *versus* expected frequencies. Descriptive statistics were computed for all quantitative traits using tree-level phenotypic means. For each trait, the number of observations, mean, standard deviation, minimum, maximum, and coefficient of variation were calculated to summarize the extent of phenotypic variability. Pairwise Pearson correlation coefficients were calculated among all quantitative traits using tree-level phenotypic means. For each trait pair, we estimated Pearson’s correlation coefficient (r), its associated p-value, and 95% confidence intervals. 

Hierarchical clustering was performed using the unweighted pair group method with arithmetic mean (UPGMA) applied to distance matrices computed from principal component (PC) scores derived from standardized (z-score) quantitative trait values. Principal component analysis (PCA) was conducted prior to clustering to reduce multicollinearity among correlated traits and to ensure that distance calculations were based on orthogonal components, thereby minimizing redundancy-driven distortion of phenotypic dissimilarities. To ensure analytical robustness, clustering performance was systematically evaluated across alternative numbers of retained PCs (2–5), distance metrics (Euclidean and Manhattan), and cluster partitions (K = 2–4). Model selection was guided by average silhouette width, cophenetic correlation and bootstrap stability. Silhouette analysis consistently supported *K* = 2, whereas higher K values produced small or weakly supported partitions. Accordingly, a two-cluster solution based on UPGMA with Euclidean distance and two retained PCs was selected as the most parsimonious and statistically supported representation of phenotypic structure.

Multi-trait genotype selection was performed using the multi-trait genotype–ideotype distance index (MGIDI), based on fruit weight (FWt), pulp content (PC), and total soluble solids (TSS), with their higher values considered desirable. Data were standardized prior to analysis. An ideotype was defined by maximum desirability after rescaling traits to a 0–100 scale, and genotypes were ranked by their Euclidean distance to this ideotype in factor space, with lower MGIDI values indicating closer alignment.

Fruit biochemical traits were analysed using linear mixed-effects models to obtain genotype-level best linear unbiased predictors (BLUPs). Measurements from within-tree fruit subsamples were used to estimate residual (within-tree) variation, while genotype was treated as a random effect. The model fitted was:

\begin{eqnarray*}{y}_{ij}=\mu +{g}_{i}+{}_{ij} \end{eqnarray*}

where *y*_ij_ is the observed value of the trait measured on the *j*th fruit subsample from the *i*th genotype (tree), µis the overall (grand) mean of the trait, *g*_i_ is the random effect of genotype *i*, and *ɛ*_ij_ is the residual error capturing within-tree fruit-to-fruit and measurement variation. Because each genotype was represented by a single tree, environmental adjustment was limited to within-tree variation. Accordingly, BLUPs represent genotype-specific deviations from the overall mean after accounting for within-tree residual variation and should be interpreted as adjusted phenotypic performance rather than genetic values. All preceding analyses were conducted in the R statistical environment (*v.* 4.5.1).

## Results

### Qualitative traits

Jamun genotypes differed significantly (*p* < 0.05) for different qualitative traits (Table 1). Tree growth habit was mainly spreading (54.47%), followed by semi-spreading (36.59%) and upright (8.94%) (*χ*^2^ = 38.83, *p* < 0.001). Tree foliage was sparse in 59.35% of the genotypes, and dense in 40.65% (*χ*^2^ = 4.30, *p* = 0.038). Leaf apex was acuminate in all genotypes, except Lk-31 in which it was acute (*χ*^2^ = 119.03, *p* < 0.001). Leaf base was predominantly acute (84.55%), while 15.45% of the genotypes had a round leaf base (*χ*^2^ = 58.74, *p* < 0.001). A similar distribution was observed for mature leaf colour, with green leaves in 84.55% of genotypes and dark green in 15.45% (*χ*^2^ = 58.74, *p* < 0.001). The mature fruit colour was predominantly purple red (50.41%) followed by purple black (32.52%) and dark purple (17.07%) (*χ*^2^ = 20.54, *p* < 0.001). Fruit shape was mainly elliptic (40.65%) or oblong (40.65%), while 17.89% of genotypes bore round fruits (*χ*^2^ = 20.54, *p* < 0.001). Fruit apex was mainly flat (73.98%), with depressed (8.13%) and round (17.89%) forms occurring less frequently (*χ*^2^ = 93.22, *p* < 0.001). Fruit stalk end was predominantly flattened (60.16%), followed by nipple-shaped (21.14%) (*χ*^2^ = 93.22, *p* < 0.001). Pulp colour was either cream-white (41.46%) or purple-white (58.54%); these two states did not differ significantly among genotypes (*χ*^2^ = 3.59, *p* = 0.058). Most genotypes exhibited medium petiole length (86.18%), and a high leaf length-to-width ratio was observed in 73.98% of the genotypes (Table 1).

**Table 1:** Frequency distribution of qualitative morphological descriptors among 123 jamun genotypes surveyed across seven districts of Uttar Pradesh, India during 2022–2024.

Trait	States	Frequency	Proportion (%)	*χ* ^2^	*p*
Tree growth habit	Spreading (3)	67	54.47	38.83	<0.001
	Semi-spreading (5)	45	36.59		
	Upright (7)	11	8.94		
Tree foliage	Sparse (1)	73	59.35	4.3	0.038
	Dense (3)	50	40.65		
Leaf apex	Acute (3)	1	0.81	119.03	<0.001
	Acuminate (7)	122	99.19		
Leaf base	Acute (3)	104	84.55	58.74	<0.001
	Round (7)	19	15.45		
Mature leaf color	Green (3)	104	84.55	58.74	<0.001
	Dark green (5)	19	15.45		
Mature fruit color	Purple red (1)	62	50.41	20.54	<0.001
	Dark purple (2)	21	17.07		
	Purple black (3)	40	32.52		
Mature fruit shape	Oblong (3)	50	40.65	55.37	<0.001
	Elliptic (5)	50	40.65		
	Ovoid (7)	1	0.81		
	Round (9)	22	17.89		
Mature fruit apex	Flat (3)	91	73.98	93.22	<0.001
	Depressed (5)	10	8.13		
	Round (7)	22	17.89		
Mature fruit stalk end	Nipple-shaped (3)	26	21.14	39.95	<0.001
	Flattened (5)	74	60.16		
	Depressed (7)	23	18.7		
Mature fruit pulp color	Cream white (1)	51	41.46	3.59	0.058ns
	Purple white (9)	72	58.54		
Petiole length	Short (3)	11	8.94	154.88	<0.001
	Medium (5)	106	86.18		
	Long (7)	6	4.88		
Leaf length: width ratio	Low (3)	32	26.02	28.30	<0.001
	High (7)	91	73.98		

**Notes.**

Observed frequencies, proportions (%), and chi-square (*χ*^2^) goodness-of-fit tests are shown for deviations from equal state distribution. Significant *χ*^2^ values (*p* < 0.001) indicate heterogeneous expression of categorical traits; ns denotes non-significant results (*p* > 0.05).

Numbers in parentheses denote numerical codes assigned to qualitative trait states for analysis.

### Leaf and fruit quantitative traits

The descriptive statistics for different leaf and fruit parameters are presented in Table 2, and tree-level (genotype) means are provided in Table S2. Leaf length varied between 11.02 cm (Sd-4) and 22.31 cm (Lk-8), with an average of 15.03 cm. Genotypes Lk-20, Lk-21, Lk-22, and Lk-30 from Lucknow district had noticeably longer leaves (≥20.0 cm), whereas certain genotypes (Hr-3, Hr-4, Hr-5, Sd-4, Sd-5, Un-2, and Un-12) from other districts had much smaller leaves (≤12.0 cm). Leaf width ranged from 3.21 cm (Sd-1) to 8.38 cm (Hr-14), with an average of 6.79 cm. The average fruit length was 2.81 cm, ranging between 1.13 cm (Ay-6) and 3.77 cm (Sd-7). Comparatively longer fruits (≥3.5 cm) were also recorded in Lk-23, Lk-26, Lk-29, Hr-1, Un-6, and Gn-10. Fruit diameter ranged from 0.85 cm (Lk-4) to 2.84 cm (Lk-7). Jamun genotypes exhibited pronounced variation in fruit weight, ranging between 2.04 g (Sd-4) and 17.66 g (Sd-7). Exceptionally low fruit weight (≤5.0 g) was recorded in Lk-10, Sd-1, Sd-2, and Un-4, whereas markedly higher fruit weight (≥13.0 g) was observed in Br-17, Gn-10, Gn-11, Hr-1, Lk-11, Lk-23, Lk-30 and Un-6. Seed length ranged between 0.82 cm (Ay-6) and 2.46 cm (Gn-10), and seed diameter between 0.58 cm (Ay-6) and 1.53 cm (Ay-4). Seed weight also varied remarkably, ranging from 0.39 g (Hr-15) to 2.39 g (Sd-5). The lowest pulp content (61.11%) was recorded in Sd-4 and the highest (94.38%) in Hr-15. Certain genotypes including Br-2, Br-6, Hr-1, Hr-9, Hr-11, Lk-13, Lk-24, Sd-7 and Un-8, also exhibited very high pulp content (≥90.0%). The pulp: seed ratio ranged between 1.60 (Sd-4) and 18.85 (Hr-15), and total soluble solids between 10.64° Brix (Lk-2) and 19.96° Brix (Lk-12).

**Table 2:** Descriptive statistics for quantitative morphological traits of 123 jamun genotypes surveyed across seven districts of Uttar Pradesh, India, during 2022–2024. Mean values represent averages of ten within-tree leaf and fruit subsamples.

**Trait/statistic**	**Mean**	**Minimum**	**Maximum**	**CV (%)**
Leaf length (cm)	15.03	11.02	22.31	15.50
Leaf width (cm)	6.79	3.21	8.38	10.70
Fruit length (cm)	2.81	1.13	3.77	15.30
Fruit diameter (cm)	2.03	0.85	2.84	13.51
Fruit weight (g)	8.74	2.04	17.66	29.30
Seed length (cm)	1.97	0.82	2.46	12.31
Seed diameter (cm)	0.93	0.58	1.53	15.61
Seed weight (g)	1.28	0.39	2.39	26.59
Pulp content (%)	84.44	61.12	94.38	5.84
Pulp: seed ratio	6.35	1.60	18.85	39.33
Total soluble solids (^∘^Brix)	13.75	10.64	19.96	15.24

**Notes.**

CV coefficient of variation (%)

### Fruit biochemical traits

The best linear unbiased predictor (BLUP) estimates indicated considerable variation among the 15 jamun genotypes for fruit biochemical traits (Table 3), reflecting substantial phenotypic diversity for quality-related attributes. Genotypes such as Gn-10, Gn-11 and Br-17 showed positive deviations for several desirable traits, including higher TSS, ascorbic acid and anthocyanin content, suggesting their potential as promising sources for improving fruit quality. In contrast, some genotypes including Br-13 and Br-15 showed below-average performance for multiple biochemical traits. Overall, the contrasting biochemical profiles highlight opportunities for trait-specific germplasm prioritization. However, as each genotype was represented by a single seedling-origin tree, these BLUPs should be interpreted cautiously as adjusted phenotypic performance rather than true genetic values.

**Table 3:** Best linear unbiased predictor (BLUP) estimates for fruit biochemical traits including total soluble solids (TSS, ^∘^Brix); titratable acidity (Acid, %); TSS: acid ratio (TSS: acid), juice pH (pH), ascorbic acid (Asc, mg 100 g^−^^1^ FW), total anthocyanins (Anth, mg 100 g^−^^1^ FW), total flavonoids (TF, mg CE g^−^^1^ FW), total phenols (TP, mg GAE 100 g^−^^1^ FW) and scavenging activity (SA, %) of 15 jamun genotypes collected during 2024 from different districts of Uttar Pradesh, India. Estimates are based on three within-tree fruit subsamples per genotype.

Genotype	TSS	Acid	TSS: acid	pH	Asc	Anth	TF	TP	SA
Br-13	−0.933	−0.462	4.703	0.020	−5.500	3.778	−0.116	−0.600	−5.923
Br-14	−1.142	−0.258	1.068	0.056	0.757	−31.928	−0.155	0.202	−1.840
Br-15	−0.813	0.195	−2.558	−0.035	−1.396	−23.273	0.119	−1.026	−4.148
Br-17	0.413	−0.210	2.163	0.081	5.135	20.068	0.049	0.720	2.664
Br-20	0.203	0.030	−0.553	0.020	−4.229	35.766	0.013	0.149	0.655
Gn-10	1.040	−0.193	2.627	−0.286	7.630	55.149	0.091	0.647	−1.139
Gn-11	0.741	0.325	−2.203	−0.163	9.036	38.958	−0.063	0.373	1.473
Gn-14	−0.783	0.279	−3.023	−0.140	−3.115	−25.741	−0.127	−0.890	2.651
Gn-15	−0.185	−0.032	−0.403	−0.035	−12.334	−15.507	0.021	0.888	4.991
Gn-18	−1.441	0.056	−2.139	0.018	5.991	−38.740	0.122	−0.369	−1.513
Un-5	0.532	0.163	−1.316	0.038	−5.555	9.372	0.024	0.584	1.996
Un-6	0.263	−0.368	4.469	0.070	7.391	28.427	−0.043	0.839	0.831
Un-8	0.861	0.441	−2.735	0.107	−0.501	−38.444	0.044	−0.706	−0.984
Un-13	0.532	0.082	−0.701	0.209	2.689	−29.296	−0.094	−0.306	4.339
Un-14	0.712	−0.048	0.600	0.038	−5.999	11.413	0.114	−0.504	−4.054

**Notes.**

Because each genotype was represented by a single seedling-origin tree, environmental adjustment was limited to within-tree residual variation; BLUPs should therefore be interpreted as adjusted phenotypic performance rather than genetic or breeding values. Positive and negative BLUP values indicate above- and below-average phenotypic performance, respectively, relative to the overall mean for each trait.

### Phenotypic corelations

Pearson’s bivariate correlations among the measured traits are presented in Table S3. Fruit size attributes were strongly and positively correlated, with fruit length (FL), fruit diameter (FD), and fruit weight (FWt) exhibiting very strong correlations (*r* = 0.71–0.88; *p* < 0.001). These traits were also positively associated with seed weight (SWt) and pulp content (PC). In contrast, total soluble solids (TSS) showed consistent negative correlations with most fruit size–and pulp-related traits, including FL, FD, FWt, SWt, and PC (*r* = −0.18 to −0.53; *P* ≤ 0.05), indicating a trade-off between sweetness and fruit size–pulp attributes. This antagonistic pattern is further supported by the strong positive association between PC and pulp–seed ratio (PSR) (*r* = 0.85; *P* < 0.001), while PSR was negatively correlated with seed-related traits. Leaf traits displayed comparatively weaker and more variable relationships with fruit characteristics.

### Cluster analysis

**Figure 1:**
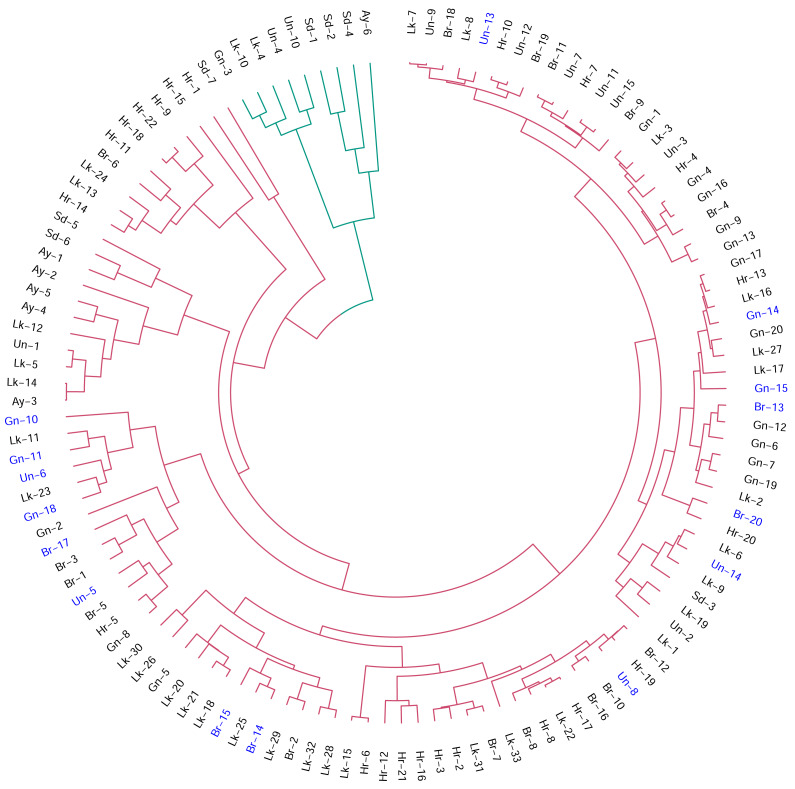
Circular dendrogram showing the hierarchical clustering of 123 jamun (*Syzygium cumini* L. Skeels) genotypes surveyed across seven districts of Uttar Pradesh, India during 2022–2024, based on standardized morphological traits. Note: Clustering was performed using Euclidean distance and the UPGMA algorithm. The analysis resolved two major phenotypic clusters, with most genotypes forming a dominant cluster (*n* = 114) and a smaller divergent cluster (*n* = 9). Genotypes selected for biochemical characterization (*n* = 15) are highlighted in blue.

**Figure 2:**
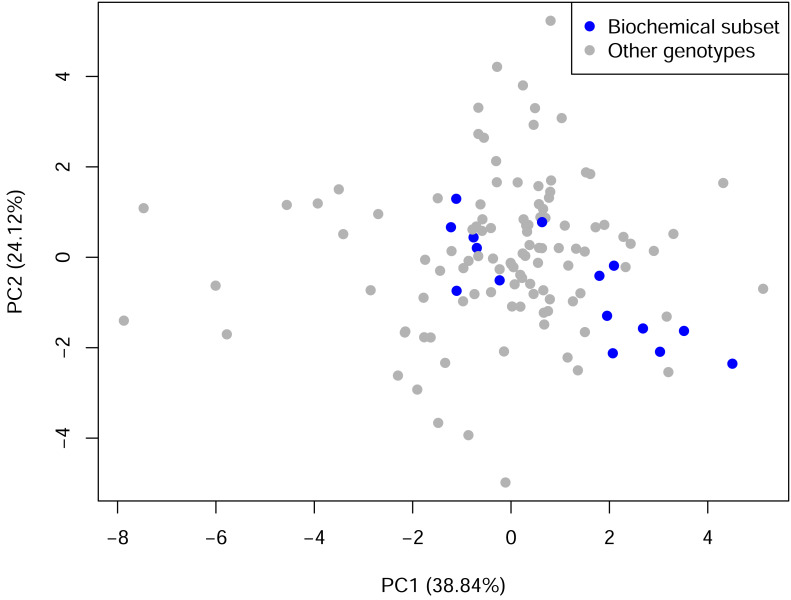
Principal component analysis (PCA) scatter plot of 123 jamun (*Syzygium cumini* (L.) Skeels) genotypes surveyed across seven districts of Uttar Pradesh, India during 2022–2024, based on standardized morphological traits. Note: The first two principal components explained 63.0% of the total phenotypic variation (PC1 = 38.84%, PC2 = 24.12%). Grey points represent the full germplasm panel, whereas blue points indicate the subset of genotypes selected for biochemical characterization, illustrating their distribution within the overall phenotypic space.

The two-cluster solution (*k* = 2) based on two principal components (PCs) and Euclidean distance emerged as the most robust configuration, yielding the highest silhouette value (0.515), strong cophenetic correlation (0.765) and high bootstrap support (0.97 and 0.676), thereby providing the most stable representation of phenotypic structure (Table S4). Cluster I comprised the majority of genotypes (*n* = 114) and represented the dominant phenotypic group. Cluster II contained a smaller set of genotypes (*n* = 9) that diverged earlier from the main assemblage, indicating comparatively greater phenotypic differentiation (Fig. 1). Although all biochemically characterized genotypes (*n* = 15) were positioned within Cluster I (Table S5), they clustered on relatively distinct branches compared with other genotypes in Cluster I. PCA based on standardized morphological traits further illustrated these relationships, with PC1 and PC2 together explaining 62.96% of the total variation. In the PCA space, the biochemical subset occupied a slightly shifted position within the dominant cluster (Fig. 2). PERMANOVA confirmed a modest but statistically significant centroid displacement relative to the remaining germplasm (*p* = 0.018), corresponding to approximately 61.43% of the average phenotypic dispersion radius, suggesting that the subset represents a phenotypically shifted segment of the dominant continuum.

### MGIDI-based genotype ranking

**Figure 3:**
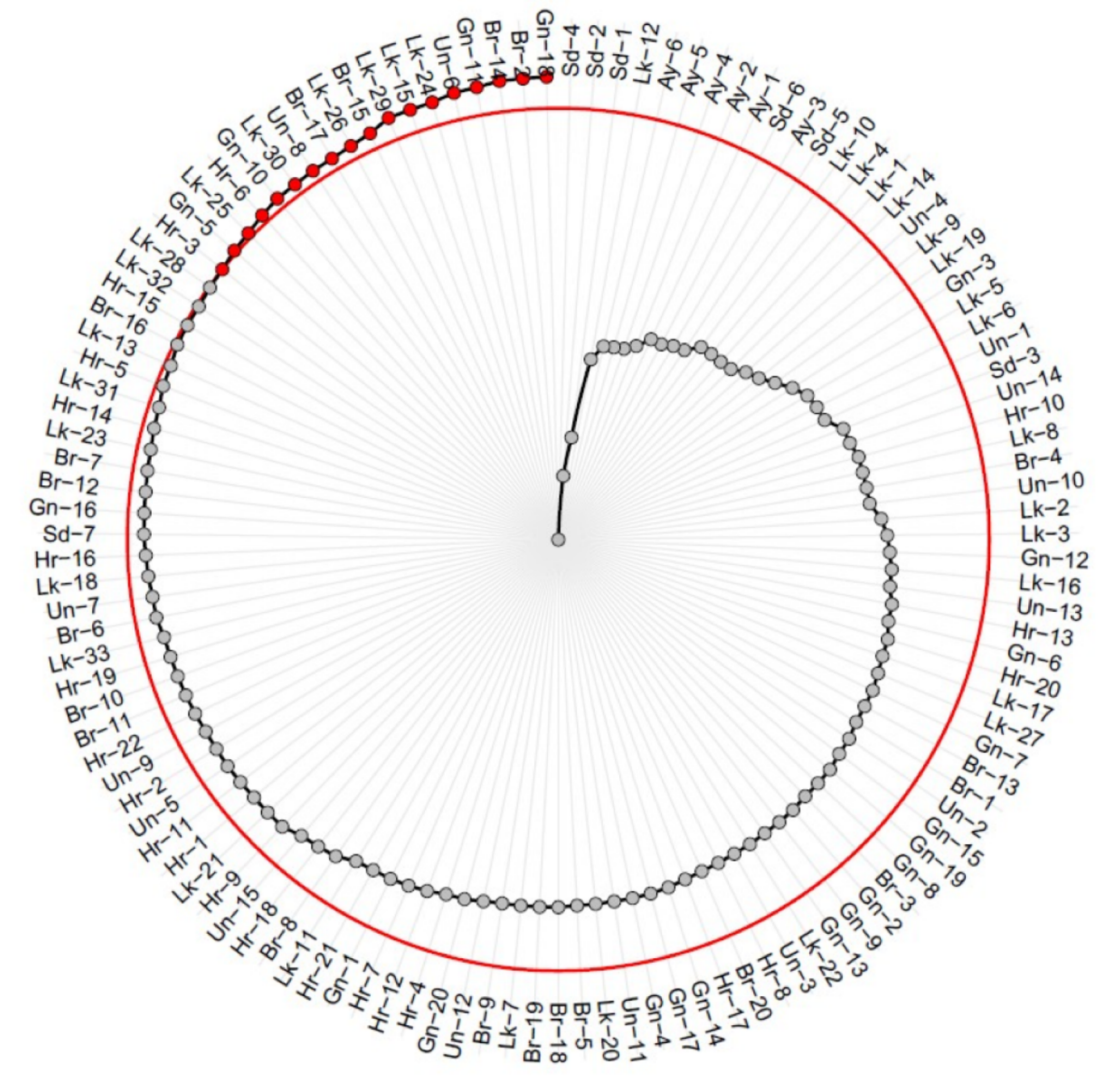
Ranking of 123 jamun (*Syzygium cumini* (L.) Skeels) genotypes surveyed across seven districts of Uttar Pradesh, India during 2022–2024 based on the Multi-trait Genotype–Ideotype Distance Index (MGIDI). Note: Selected genotypes including Gn-18, Br-2, Br-14, Gn-11 and Un-6 (red points) represent the top-performing candidates based on the multi-trait selection criterion, while grey points denote non-selected genotypes. Genotypes with lower MGIDI values are closer to the predefined ideotype and therefore considered more desirable for simultaneous improvement of key fruit traits.

The results of Principal Component Analysis (PCA), factor loadings, mean shifts under selection and MGIDI rankings are summarized in Table S6, while the genotype–ideotype distance distribution and MGIDI-based phenotypic selection pattern are shown in Fig. 3. The first two principal components explained 89.52% of the total variation among traits (PC1 = 62.25%, PC2 = 27.28%). Factor analysis showed that FWt (FA1 = −0.73) and PC (−0.91) were strongly associated with the primary latent factor, whereas TSS (= 0.71) loaded in the opposite direction. This pattern points to a clear trade-off between fruit size and pulp traits *versus* sweetness, indicating that genotypes producing larger fruits with higher pulp content tend to have lower TSS, and vice versa. Communality values further suggest that PC was best explained by this factor (0.82), followed by FWt (0.54) and TSS (0.51), indicating that FA1 captures a substantial share of variation in these key quality attributes. Based on MGIDI values, the top-performing genotypes were Gn-18, Br-2, Br-14, Gn-11 and Un-6. These genotypes exhibited favourable combined expression of FWt and PC, while maintaining acceptable TSS levels. Mean shift analysis showed a strong increase in FWt (+37.5%) and a moderate increase in PC (+4.8%), but this was accompanied by a small decline in TSS (−7.9%). Together, these results highlight a clear trade-off between these traits. It is, however, important to note that the reported improvements in fruit weight and pulp content in the present study reflect the phenotypic advantage of the selected genotypes over the population mean and should not be interpreted as a predicted genetic response to selection.

## Discussion

Despite considerable economic potential, jamun remains an underutilized fruit crop in India. Jamun fruits are still largely obtained from traditionally managed seedling populations on fallow and forest lands (Tripathi et al., 2025). The development of superior jamun varieties depends to a considerable extent on the effective characterization and utilization of available germplasm, with particular emphasis on phenotypic diversity in fruit weight, pulp content and other key pomological traits (Sane, Rekha & Tripathi, 2025). Previous studies have mostly evaluated only a limited number of jamun genotypes (Singh et al., 2022), and systematic assessments of diversity in seedling populations of jamun from Uttar Pradesh, India remain scarce. Because population size strongly influences selection intensity and response (Sinha et al., 2021), the present study was carried out to determine phenotypic variability and identify superior genotypes from a large set of 123 jamun genotypes.

Morphological characterization is typically the first step in assessing phenotypic variability, and leaf and fruit descriptors remain among the most informative criteria for distinguishing species and varieties (Atefe, Kambiz & Javad, 2015; Wang et al., 2022). In the present study, jamun genotypes exhibited considerable diversity across qualitative and quantitative attributes of trees, leaves and fruits (Prakash, Maurya & Singh, 2010; Singh et al., 2020a; Singh et al., 2020b; Khadivi et al., 2023; Kumar et al., 2025; Sharma et al., 2023). Notably, tree growth habit has direct implications for orchard management. Spreading canopies are generally associated with vigorous tree growth (Singh et al., 2025), whereas erect types may be more suitable for high-density systems, albeit with potential challenges in pruning, canopy management and harvesting (Latheef & Kavino, 2018). A descriptive comparison suggested that certain qualitative traits tended to be more frequent in specific districts. However, these patterns are observational and were not formally tested for statistical association. For example, genotypes from Lucknow predominantly exhibited spreading to semi-spreading tree growth; those from Gonda mainly showed purple-red to dark purple mature fruit colour. Likewise, genotypes from Hardoi consistently displayed a flat fruit apex, with nearly all also having a flattened fruit stalk end.The tested jamun genotypes exhibited marked differences across all leaf and fruit quantitative traits. The ranges observed for leaf length (11.02–22.31 cm) and width (3.21–8.38 cm) were comparable to those reported by Kumar et al. (2025) but differed from values documented by Anushma & Sane (2018) and Soundarya et al. (2024), likely reflecting differences in experimental conditions. Larger leaves may confer enhanced photosynthetic capacity and greater assimilate availability for fruit development (Dissanayake et al., 2022). Fruit weight (2.04–17.66 g) and seed weight (0.39–2.39 g) spanned considerably broader ranges than previously reported (Singh et al., 2020a; Singh et al., 2020b; Anushma & Sane, 2018). The wide phenotypic diversity observed in the present study underscores the potential for identifying and prioritizing jamun genotypes with larger fruits and higher pulp content (Morariu et al., 2025). Jamun genotypes can be classified into low (<80%), medium (80–90%), and high (>90%) pulp-content categories (PPVFRA, 2025). Most genotypes in this study fell into the medium class (75.61%), with relatively few in the low (17.07%) or high (7.32%) categories. Although high pulp content is a desirable trait, fruit weight remains equally important for improving edible portion and market value (Chandra et al., 2020; Singh et al., 2020a; Singh et al., 2020b). Reports of seedless jamun types exist, but their extremely small fruit size (<1 g) limits commercial relevance (Tripathi et al., 2025). The pulp: seed ratio observed here (1.60–18.85) exceeded earlier ranges reported in India (Plathia et al., 2018; Raja, 2020) and elsewhere (ud Din et al., 2020), indicating broader phenotypic dispersion. TSS also varied widely, reflecting meaningful differences in sweetness and palatability (Singh & Swamy, 2021). Overall, the magnitude of morphological variability observed reflects the underexploited potential of jamun seedling populations and reinforces the need to conserve diverse germplasm to support future breeding efforts (Karunakaran et al., 2025; Nadarajan et al., 2021).

In this study, several quantitative traits showed coefficients of variation (CV) > 15%, indicating substantial overall variability among jamun genotypes (Khadivi et al., 2023). Overall, pulp: seed ratio (CV = 39.33%), fruit weight (29.30%), and seed weight (26.59%) were the most variable traits, indicating phenotypic diversity amenable to prioritizing promising genotypes.

The 15 selected jamun genotypes displayed considerable variability in biochemical traits, consistent with previous reports (Tayade, 2019; ud Din et al., 2020) and likely reflecting distinct phenotypic characteristics (Shahnawaz & Sheikh, 2011). Ascorbic acid (Singh & Kaur, 2016; Rajangam & Sankar, 2022) and anthocyanin (Ghosh et al., 2017) contents fell within or above published ranges for jamun. Total phenolics and flavonoids tended to exceed earlier estimates for jamun (Kumar et al., 2025), likely because a diverse germplasm set was assessed in the present study. BLUPs highlighted a small subset of genotypes combining high TSS, favourable TSS: acid balance, and elevated ascorbic acid and anthocyanin contents, indicating clear opportunities to select for genotypes with enhanced nutritional properties (Cruet-Burgos, Morris & Rhodes, 2023). However, because each genotype was represented by a single seedling-origin tree, the BLUPs should be interpreted cautiously as adjusted phenotypic performance rather than true genetic values.

The UPGMA dendrogram based on Euclidean distances grouped the 123 jamun genotypes into two clusters. Cluster 1 contained most genotypes, whereas Cluster two comprised nine genotypes. The relatively high silhouette value (0.515), together with strong cophenetic correlation (0.765) and substantial bootstrap support (97% and 67.6%) for the two-cluster solution (*k* = 2) indicates that this clustering provides a reliable representation of the underlying phenotypic structure (Singh et al., 2022). The clustering of genotypes from different districts within the same clusters suggests that geographic origin does not always correspond to phenotypic divergence, likely due to shared ancestry or inter-district seed exchange (Norouzi et al., 2017). From a germplasm management perspective, such clustering highlights the presence of phenotypic redundancy among accessions, indicating that representative genotypes from each cluster could be prioritized for conservation, evaluation and future breeding programs. Nevertheless, integrating molecular characterization may assist in more precise characterization and germplasm prioritization (Sharma et al., 2023). The MGIDI provides an integrative framework for multi-trait evaluation, allowing complex trait combinations to be assessed simultaneously and genotypes to be ranked according to their proximity to an ideotype (Debnath et al., 2024). In this study, MGIDI effectively discriminated among genotypes, identifying Gn-18, Br-2, Br-14, Gn-11 and Un-6 as the most promising candidates (Wang et al., 2024). These selections reflected a favourable balance of high fruit weight and pulp content while maintaining acceptable TSS levels. The accompanying selection differential analysis further indicated a strong positive response for FWt (+37.5%) and a moderate gain for PC (+4.81%), with a modest but expected decline in TSS (−7.88%) as a trade-off.

## Conclusions

The present study highlights unexplored phenotypic variability among seedling-origin jamun trees sampled across seven districts of Uttar Pradesh, India, with the extent of variation appearing to be trait-specific. Several ideotype-aligned genotypes were identified as promising under the studied conditions; however, because observations were recorded from single trees during a single year, the results should be interpreted cautiously and validated through replicated multi-environment trials before broader generalization. Future efforts incorporating wider germplasm sampling, additional environments and molecular marker-based approaches will help extend the applicability of these findings and improve selection efficiency in jamun improvement programs.

##  Supplemental Information

10.7717/peerj.21302/supp-1 Supplemental Information 1 Codebook

10.7717/peerj.21302/supp-2 Supplemental Information 2 R Scripts used in statistical analysis

10.7717/peerj.21302/supp-3 Supplemental Information 3 Supplementary tables and Figures

10.7717/peerj.21302/supp-4 Supplemental Information 4 Raw data

## Abbreviations

BLUP: Best Linear Unbiased Predictor
CI: Confidence Interval
CV: Coefficient of Variation
DPPH: 2,2-Diphenyl-1-picrylhydrazyl
FA: Factor Analysis
FD: Fruit Diameter
FL: Fruit Length
FW: Fresh Weight
FWt: Fruit Weight
GAE: Gallic Acid Equivalents
LL: Leaf Length
LW: Leaf Width
MGIDI: Multitrait Genotype–Ideotype Distance Index
PC: Pulp Content
PSR: Pulp-to-Seed Ratio
QE: Quercetin Equivalent(s)
SD: Seed Diameter
SE: Standard Error
SL: Seed length
SWt: Seed Weight
TSS: Total Soluble Solids
TSS: acid: TSS acid ratio
UPGMA: Unweighted Pair Group Method with Arithmetic Mean

## Additional Information and Declarations

### Data Availability

The following information was supplied regarding data availability: The raw data is available in the Supplemental File.
