# Supplementary material for: Assessing phenotypic diversity and trait relationships within (Syzygium cumini (L.) Skeels) using morpho-biochemical traits
Source: PeerJ. 2026 May 25;14:e21302. doi: 10.7717/peerj.21302 (PMC13218339; doi:10.7717/peerj.21302)
Supplement: Supplemental Information 2 [file peerj-14-21302-s002.docx]

**R Syntax for frequency distribution and chi-square goodness-of-fit analysis of qualitative phenotypic traits**

library(dplyr)

library(tidyr)

Quali[, -1] <- lapply(Quali[, -1], factor)

traits_quali <- Quali[, -1]

qual_summary <- lapply(names(traits_quali), function(tr) {

x <- traits_quali[[tr]]

tab <- table(x)

chi <- chisq.test(tab)

data.frame(

Trait = tr,

Category = names(tab),

Frequency = as.vector(tab),

Proportion = round(as.vector(prop.table(tab)), 3),

Chi_sq = round(chi$statistic, 3),

df = chi$parameter,

p_value = round(chi$p.value, 4)

)

})

qual_summary <- bind_rows(qual_summary)

qual_summary

**R syntax for descriptive statistics**

library(dplyr)

library(tidyr)

traits <- Means %>% select(where(is.numeric))

desc_stats <- traits %>%

summarise(

across(

everything(),

list(

n = ~sum(!is.na(.)),

mean = ~mean(., na.rm = TRUE),

sd = ~sd(., na.rm = TRUE),

min = ~min(., na.rm = TRUE),

max = ~max(., na.rm = TRUE),

cv = ~sd(., na.rm = TRUE)/mean(., na.rm = TRUE) * 100

),

.names = "{.col}_{.fn}"

),

.groups = "drop"

) %>%

pivot_longer(

cols = everything(),

names_to = c("Trait", "Statistic"),

names_sep = "_",

values_to = "Value"

) %>%

pivot_wider(

names_from = Statistic,

values_from = Value

) %>%

arrange(Trait)

desc_stats

**R syntax for Internal clustering diagnostics for Ward’s method (k = 2–10)**

library(readxl)

library(cluster)

library(fpc)

## -----------------------------

## 1. Data preparation

## -----------------------------

Means <- read_excel(

"C:/Users/Anshuman Singh/Desktop/Final_10-12-2025/Revisions_Jan_2026/Means.xlsx",

col_types = c("text", "text", rep("numeric", 11))

)

df <- as.data.frame(Means)

rownames(df) <- df[, 1]

Xz <- scale(df[, -1])

## -----------------------------

## 2. Ward clustering

## -----------------------------

d <- dist(Xz, method = "euclidean")

hc <- hclust(d, method = "ward.D2")

dist_mat <- as.matrix(d)

## -----------------------------

## 3. CH + BW ratio for k = 2–10

## -----------------------------

k_vals <- 2:10

results <- data.frame(

k = k_vals,

Calinski_Harabasz = NA,

BW_ratio = NA

)

for (i in seq_along(k_vals)) {

k <- k_vals[i]

cl <- cutree(hc, k)

## CH index

results$Calinski_Harabasz[i] <- cluster.stats(d, cl)$ch

## Mean within-cluster distance

within_vals <- sapply(unique(cl), function(g) {

idx <- which(cl == g)

mean(dist_mat[idx, idx][upper.tri(dist_mat[idx, idx])])

})

mean_within <- mean(within_vals)

## Mean between-cluster distance

groups <- unique(cl)

between_vals <- c()

for (a in 1:(length(groups) - 1)) {

for (b in (a + 1):length(groups)) {

idx_a <- which(cl == groups[a])

idx_b <- which(cl == groups[b])

between_vals <- c(

between_vals,

mean(dist_mat[idx_a, idx_b])

)

}

}

mean_between <- mean(between_vals)

## Between / within ratio

results$BW_ratio[i] <- mean_between / mean_within

}

## -----------------------------

## 4. Print / export results

## -----------------------------

results

write.csv(

results,

file = "Ward_CH_and_BW_ratio_k2_10.csv",

row.names = FALSE

)

**R Syntax for bivariate correlations**

library(readxl)

Means <- read_excel(

"C:/Users/Anshuman Singh/Desktop/Final_10-12-2025/Revisions_Jan_2026/Means.xlsx",

col_types = c("text", "text", rep("numeric", 11))

)

traits <- Means[, sapply(Means, is.numeric)]

trait_names <- colnames(traits)

pairs <- combn(trait_names, 2)

cor_tbl <- apply(pairs, 2, function(p) {

ct <- cor.test(traits[[p[1]]], traits[[p[2]]], method = "pearson")

c(

Trait1 = p[1],

Trait2 = p[2],

r = ct$estimate,

lower_CI = ct$conf.int[1],

upper_CI = ct$conf.int[2],

p_value = ct$p.value

)

})

cor_tbl <- as.data.frame(t(cor_tbl), stringsAsFactors = FALSE)

num_cols <- c("r", "lower_CI", "upper_CI", "p_value")

for (cc in num_cols) cor_tbl[[cc]] <- as.numeric(cor_tbl[[cc]])

cor_tbl$Significance <- ifelse(

cor_tbl$p_value < 0.001, "***",

ifelse(cor_tbl$p_value < 0.01, "**",

ifelse(cor_tbl$p_value < 0.05, "*", "ns"))

)

write.csv(

cor_tbl,

"Phenotypic_correlations_Pearson_95CI_significance.csv",

row.names = FALSE

)

**R syntax for PCA-based hierarchical clustering and validation**

library(readxl)

library(cluster)

library(fpc)

Means <- read_excel(

"C:/Users/Anshuman Singh/Desktop/Final_10-12-2025/Revisions_Jan_2026/Means.xlsx",

col_types = c("text", "text", rep("numeric", 11))

)

df <- as.data.frame(Means)

genotypes <- make.unique(as.character(df$Tree_ID))

X <- df[,3:13]

Xz <- scale(X)

rownames(Xz) <- genotypes

pca <- prcomp(Xz, center = FALSE, scale. = FALSE)

results_master <- data.frame()

for (k_pc in 2:5) {

pc_scores <- pca$x[,1:k_pc]

for (dist_method in c("euclidean","manhattan")) {

d_mat <- dist(pc_scores, method = dist_method)

hc <- hclust(d_mat, method = "average")

cophenetic_r <- cor(d_mat, cophenetic(hc))

for (k in 2:4) {

clusters <- cutree(hc, k)

sil_val <- mean(silhouette(clusters, d_mat)[,3])

set.seed(123)

boot_res <- clusterboot(

pc_scores,

B = 1000,

clustermethod = hclustCBI,

method = "average",

k = k

)

boot_vals <- boot_res$bootmean

cluster_sizes <- table(clusters)

results_master <- rbind(

results_master,

data.frame(

PCs = k_pc,

Distance = dist_method,

K = k,

Cophenetic = round(cophenetic_r,3),

Silhouette = round(sil_val,3),

Boot1 = round(boot_vals[1],3),

Boot2 = ifelse(length(boot_vals)>1, round(boot_vals[2],3), NA),

MinClusterSize = min(cluster_sizes),

MaxClusterSize = max(cluster_sizes)

)

)

}

}

}

print(results_master)

**R syntax for circular dendrogram**

library(readxl)

library(dendextend)

library(circlize)

Means <- read_excel(

"C:/Users/Anshuman Singh/Desktop/Final_10-12-2025/Revisions_Jan_2026/Means.xlsx",

col_types = c("text","text",rep("numeric",11))

)

df <- as.data.frame(Means)

genotypes <- make.unique(as.character(df$Tree_ID))

X <- df[,3:13]

Xz <- scale(X)

rownames(Xz) <- genotypes

pca <- prcomp(Xz, center = FALSE, scale. = FALSE)

pc_scores <- pca$x[,1:2]

d_mat <- dist(pc_scores, method="euclidean")

hc <- hclust(d_mat, method="average")

dend <- as.dendrogram(hc)

dend <- ladderize(dend)

dend <- color_branches(dend, k=2)

dend <- set(dend, "labels_cex", 0.6)

bio_subset <- c(

"Br-13","Br-14","Br-15","Br-17","Br-20",

"Un-5","Un-6","Un-8","Un-13","Un-14",

"Gn-10","Gn-11","Gn-14","Gn-15","Gn-18"

)

labels_colors(dend) <- ifelse(labels(dend) %in% bio_subset,"blue","black")

pdf("Jamun_circular_dendrogram.pdf", width=10, height=10)

circos.clear()

circos.par(start.degree=90, gap.degree=2)

circlize_dendrogram(dend, labels_track_height=0.30)

dev.off()

**R script for PCA visualization and PERMANOVA-based centroid displacement analysis**

library(readxl)

library(vegan)

Means <- read_excel(

"C:/Users/Anshuman Singh/Desktop/Final_10-12-2025/Revisions_Jan_2026/Means.xlsx",

col_types = c("text","text",rep("numeric",11))

)

df <- as.data.frame(Means)

genotypes <- make.unique(as.character(df$Tree_ID))

X <- df[,3:13]

Xz <- scale(X)

rownames(Xz) <- genotypes

pca <- prcomp(Xz, center = FALSE, scale. = FALSE)

var_explained <- (pca$sdev^2)/sum(pca$sdev^2)

pc_scores <- as.data.frame(pca$x[,1:2])

pc_scores$Genotype <- rownames(pc_scores)

bio_subset <- c(

"Br-13","Br-14","Br-15","Br-17","Br-20",

"Un-5","Un-6","Un-8","Un-13","Un-14",

"Gn-10","Gn-11","Gn-14","Gn-15","Gn-18"

)

pc_scores$Group <- ifelse(pc_scores$Genotype %in% bio_subset,

"Biochemical subset",

"Other genotypes")

d_mat <- dist(pc_scores[,1:2], method="euclidean")

group <- pc_scores$Group

adonis_res <- adonis2(d_mat ~ group)

centroid_all <- colMeans(pc_scores[,1:2])

centroid_bio <- colMeans(pc_scores[pc_scores$Group=="Biochemical subset",1:2])

centroid_shift <- sqrt(sum((centroid_bio-centroid_all)^2))

mean_dispersion <- mean(sqrt(rowSums((pc_scores[,1:2]-centroid_all)^2)))

percent_shift <- (centroid_shift/mean_dispersion)*100

print(round(var_explained[1:2]*100,2))

print(adonis_res)

print(round(percent_shift,2))

pdf("C:/Users/Anshuman Singh/Desktop/PCA_scatter_plot.pdf", width=7, height=6)

plot(pc_scores$PC1, pc_scores$PC2,

col=ifelse(group=="Biochemical subset","blue","grey70"),

pch=19,

xlab=paste0("PC1 (",round(var_explained[1]*100,2),"%)"),

ylab=paste0("PC2 (",round(var_explained[2]*100,2),"%)"))

legend("topright",

legend=c("Biochemical subset","Other genotypes"),

col=c("blue","grey70"),

pch=19)

dev.off()

**R syntax for MGIDI**

library(readxl)

library(dplyr)

library(metan)

Means <- read_excel(

"C:/Users/Anshuman Singh/Desktop/Final_10-12-2025/Revisions_Jan_2026/Means.xlsx",

col_types = c("text","text",rep("numeric",11))

)

traits <- Means %>% select(FWt, PC, TSS)

mgidi_data <- Means %>% select(Tree_ID, FWt, PC, TSS)

res_mgidi <- mgidi(

mgidi_data,

ideotype = c("h","h","h")

)

mgidi_rank <- res_mgidi$MGIDI %>% arrange(MGIDI)

write.csv(mgidi_rank, "MGIDI_full_ranking.csv", row.names = FALSE)

mgidi_top10 <- mgidi_rank %>% slice(1:10)

write.csv(mgidi_top10, "MGIDI_top10.csv", row.names = FALSE)

pca <- prcomp(traits, scale. = TRUE)

eig <- pca$sdev^2

var <- eig / sum(eig) * 100

cum <- cumsum(var)

pca_summary <- data.frame(

Component = paste0("PC", 1:length(eig)),

Eigen_value = round(eig, 2),

Variance_percent = round(var, 2),

Cumulative_percent = round(cum, 2)

)

write.csv(pca_summary, "PCA_summary.csv", row.names = FALSE)

traits_fa <- scale(traits) %>% na.omit()

fa <- factanal(traits_fa, factors = 1, rotation = "none")

load <- as.matrix(fa$loadings)

fa_table <- data.frame(

Trait = rownames(load),

FA1_Loading = round(load[,1], 2),

Communality = round(rowSums(load^2), 2)

)

write.csv(fa_table, "FA_loadings_communalities.csv", row.names = FALSE)

Xo <- colMeans(traits)

selected <- mgidi_top10$Genotype

Xs <- Means %>%

filter(Tree_ID %in% selected) %>%

summarise(across(c(FWt, PC, TSS), mean)) %>%

unlist()

SD <- Xs - Xo

SDp <- (SD / Xo) * 100

sel_diff <- data.frame(

Trait = names(Xo),

Xo = round(Xo, 2),

Xs = round(Xs, 2),

SD = round(SD, 2),

SD_percent = round(SDp, 1),

Goal_met = ifelse(SD > 0, "Yes", "No")

)

write.csv(sel_diff, "Selection_differential.csv", row.names = FALSE)

cor_table <- round(cor(traits), 2)

write.csv(cor_table, "Trait_correlations.csv")

pdf("MGIDI_circular_plot.pdf", width = 8, height = 8)

plot(

res_mgidi,

index = "MGIDI",

select = 10,

col.select = "red",

col.nonselect = "grey70"

)

dev.off()

**R Syntax for BLUPs (fruit biochemical traits)**

library(readxl)

library(lme4)

library(dplyr)

Biochem <- read_excel(

"C:/Users/Anshuman Singh/Desktop/Final_10-12-2025/Revisions_Jan_2026/Biochem.xlsx",

col_types = c("text","text","numeric","numeric","numeric",

"numeric","numeric","numeric","numeric","numeric","numeric")

)

traits <- c("TSS","Acid","TA","pH","Asc","Anth","TF","TP","SA")

blup_list <- lapply(traits, function(tr){

m <- lmer(as.formula(paste(tr, "~ 1 + (1 | Gen)")), data = Biochem)

out <- ranef(m)$Gen

colnames(out) <- tr

out

})

BLUP_table <- as.data.frame(Reduce(cbind, blup_list))

BLUP_table$Gen <- rownames(BLUP_table)

BLUP_table <- BLUP_table %>% relocate(Gen)

View(BLUP_table)

write.csv(BLUP_table, "Table6_BLUPs_Biochem.csv", row.names = FALSE)
